# Usage and Acceptability of the iBobbly App: Pilot Trial for Suicide Prevention in Aboriginal and Torres Strait Islander Youth

**DOI:** 10.2196/14296

**Published:** 2020-12-01

**Authors:** Joseph Tighe, Fiona Shand, Kathy McKay, Taylor-Jai Mcalister, Andrew Mackinnon, Helen Christensen

**Affiliations:** 1 Black Dog Institute Sydney Australia; 2 Men's Outreach Service Broome Australia; 3 Tavistock and Portman NHS Foundation Trust London United Kingdom; 4 Public Health, Policy and Systems Institute of Population Health University of Liverpool Liverpool United Kingdom; 5 Centre for Health Informatics Australian Institute of Health Innovation Macquarie University Sydney Australia

**Keywords:** mHealth, suicide, depression, eHealth, Indigenous, Aboriginal, First Nations, mental health, suicide ideation, apps

## Abstract

**Background:**

The proliferation of mental health apps purporting to target and improve psychological wellbeing is ever-growing and also concerning: Few apps have been rigorously evaluated, and, indeed, the safety of the vast majority of them has not been determined. Over 10,000 self-help apps exist but most are not used much after being downloaded. Gathering and analyzing usage data and the acceptability of apps are critical to inform consumers, researchers, and app developers.

**Objective:**

This paper presents pilot usage and acceptability data from the iBobbly suicide prevention app, an app distributed through a randomized controlled trial.

**Methods:**

Aboriginal and Torres Strait Islander participants from the Kimberley region of Western Australia completed a survey measuring their technology use in general (n=13), and data on their experiences with and views of the iBobbly app were also collected in semistructured interviews (n=13) and thematically analyzed. Finally, engagement with the app, such as the number of sessions completed and time spent on various acceptance-based therapeutic activities, was analyzed (n=18). Both groups were participants in the iBobbly app pilot randomized controlled trial (n=61) completed in 2015.

**Results:**

Regression analysis indicated that app use improved psychological outcomes, although only minimally, and effects were not significant. However, results of the thematic analysis indicated that the iBobbly app was deemed effective, acceptable, and culturally appropriate by those interviewed.

**Conclusions:**

There is a scarcity of randomized controlled trials and eHealth interventions in Indigenous communities, while extremely high rates of psychological distress and suicide persist. In this environment, studies that can add evidence from mixed-methods approaches are important. While the regression analysis in this study did not indicate a significant effect of app use on psychological wellbeing, this was predictable considering the small sample size (n=18) and typically brief app use. The results on engagement with the iBobbly app were however positive. This study showed that Indigenous youth are early and frequent users of technology in general, and they regarded the iBobbly app to be culturally safe and of therapeutic value. Qualitative analyses demonstrated that iBobbly app use was associated with self-reported improvements in psychological wellbeing, mental health literacy, and reductions in shame. Importantly, participants reported that they would recommend other similar apps if available to their peers.

## Introduction

Aboriginal and Torres Strait Islander (herein referred to as Indigenous Australian) suicide rates continue to be recorded at extraordinarily high levels, with rates of suicide among Indigenous Australians twice that of the non-Indigenous population [1]. This paper focuses on Indigenous youth, whose suicide rates are 4 times those of other Australian youth [1]. Indigenous suicide and suicidal behavior are different from those of non-Indigenous Australians and influenced by poverty and a range of historical, sociocultural, and sociopolitical factors [2,3]. Indeed, the terms “mental health/illness” and much of the western biomedical terminology around suicide are of questionable value to Indigenous health experts and community members, who view mental health through their holistic interconnected model of Social and Emotional Wellbeing (SEWB) [4,5]. This collectivist understanding of health care takes a community-wide view and values connection to family, culture, ancestry, land, and spirituality as some of the factors in maintaining wellness [4,5]. Disconnection, dispossession, and ongoing trauma since colonization have contributed to the severe health inequities Indigenous people suffer today [4,6,7]. These social determinants of health are contributing to the disturbingly high rates of Indigenous youth suicide evident over recent decades [4,6,7].

Rates of suicide among Indigenous people are even higher in regional and remote Australia where fewer health services are available [1]. Access to SEWB services deemed culturally safe is difficult in these vast and sparsely populated regions, although Indigenous-controlled health organizations attempt to address this [7,8]. Despite the remoteness of many communities, the vast majority have effective telecommunication systems including mobile phone coverage. Varying levels of trust of non-Indigenous health services and health researchers lead to challenges in establishing purposeful collaborations with Indigenous communities [9]. Effective research with Indigenous peoples requires significant investment in relationship building, community approvals, and additional ethical clearances [9].

Unsurprisingly, co-design and effective partnerships are recommended for progress [9-11], and cultural safety can be achieved through co-design and testing of SEWB interventions with end users [12-14]. Technology solutions are worth examining, as more Indigenous people are using smartphones than other Australians (70% vs 66%) and Indigenous youth are also using social media such as Facebook 20% more frequently than other Australian youth [15].

App or mobile health (mHealth) interventions have the potential to overcome some of the common barriers to help-seeking [14,16], as they are “always on” by design and available to hand without the need to attend face-to-face appointments even if the latter are available. Anonymity can be ensured through minimal sign-up, and autonomy and empowerment are supported as users choose to use the apps or not. mHealth and eHealth can serve as adjunctive or alternative treatment to traditional face-to-face services. Indigenous people have extensively reported feelings of “shame” in accessing some health services and in speaking with professionals about mental health issues [16,17]. Apps can attempt to bypass this “shame” in addition to overcoming any embarrassment or discomfort due to poor literacy through the inclusion of voiceover features [13].

The acceptability and usability of apps are critical if they are to effect change in mental health symptoms [18]. To create apps that are widely used, it is also important for researchers to understand the motivations for and pathways to participation, the capacity of apps to maintain users’ engagement, and the potential benefits of increased app use. Culturally appropriate interventions and SEWB services can assist in tackling Indigenous youth suicide [7,10,19]. The iBobbly app was developed as one such intervention [13]. Having previously reported on the effectiveness of iBobbly in reducing suicidal ideation, depression, distress, and impulsivity [12,13], the authors sought to understand the extent to which the app was used and to explore cultural appropriateness, acceptability, and therapeutic value. Featuring acceptance-based therapeutic activities and based on acceptance and commitment therapy, the app was designed and developed in partnership with local Indigenous stakeholders in the Kimberley region of north Western Australia and trialed following ethical approval between September 2013 and March 2015 [12,13]. In remote areas, it was designed to operate without internet connectivity [12,13]. This development involved the input of focus groups of Indigenous youth around content and the creation of original artwork or imagery and audio by Indigenous artists working closely with the app developers. As the first trial of an app to target suicide prevention in any population and considering the sensitivities around youth suicide, it was important for stakeholders to examine participant engagement. As a small qualitative component of the randomized controlled trial (RCT) itself, researchers had a rare opportunity to conduct interviews with a smaller group of participants. It was hoped that feedback regarding possible improvements to future versions of the app or to the research processes could be gathered. This paper addresses the following research questions:

- What was the nature of general technology use among this cohort of Indigenous youth?
- To what extent did participants find the iBobbly app acceptable, culturally appropriate, and therapeutically effective?
- Was greater time spent on the app associated with improved therapeutic outcomes?

## Methods

### Participants

Participants (n=13) were purposefully selected due to their close proximity to the research team in Broome, Western Australia and their willingness to engage. These participants were part of the larger cohort (N=61) who participated in the iBobbly app RCT in the wider region [12,13]. Indigenous people from 18 to 35 years of age were recruited if they recorded moderate scores for depression or psychological distress, with or without current suicidal ideation [12,13]. While initially targeting just those aged 18-25 years (youth), the age range was widened to aid recruitment.

### Data Collection

#### General Internet Use Survey

Data on general internet use were collected for 13 trial participants using The Measures of Technology Use survey developed by the Young and Well Cooperative Research Centre [20] (Multimedia Appendix 1). The survey contained 10 items covering frequency and duration of internet use in general, types of devices used online, and types of online activities engaged in. There were no adaptions made to the survey for the cohort interviewed.

#### Semistructured Interviews

Data were collected from semistructured interviews with the same 13 participants on their experience with using the app. The interview guide (Multimedia Appendix 2) consisted of 12 questions that were designed for a brief interview. The interview questions were reviewed and approved by Indigenous mental health professionals, and all participants were asked the same fixed, yet open-ended, questions. Of the 13 participants approached to be interviewed, all 13 agreed to take part. Interviews were not recorded, as recording Indigenous youth speaking about their own suicidality with an unfamiliar non-Indigenous researcher was regarded as potentially uncomfortable for participants.

### Procedures

Three researchers (JT, FS, and HC) developed the interview guide relating to whether the app was deemed effective, culturally appropriate, and acceptable and recorded referral pathways and personal motivations around participation. As part of the consent process for the trial, participants were asked if they were willing to be followed up. This allowed us to follow them up with both standardized measures and to invite them to be part of the qualitative interviews. JT approached participants and interviewed those who provided consent to complete the interview and the aforementioned survey. The interview was conducted face-to-face following the 6-week app trial. All notes were hand-written verbatim, verified with participants to ensure accuracy, revised if needed, and transcribed. There were few edits made to the original notes following revision by participants. In order to maintain confidentiality, participants are referred to by their transcription code: age, gender, and transcription number. For example, 18-F-3 refers to an 18-year-old female who was the third person to be interviewed. After all data were collected, two researchers (JT, KM) independently analyzed the transcripts using an inductive approach appropriate given the exploratory nature of the study. These transcripts were read several times, with KM clarifying cultural or experiential uncertainties with JT, as he had conducted the interviews. A thematic analysis was conducted in line with the approach set out by Braun and Clarke [21], where the 2 researchers systematically coded the transcripts and sorted these codes into themes. Any disagreement was discussed and resolved. It was acknowledged that, while the transcripts were not always long, the focus of the research was clear and narrow, with dense sample specificity [22]. KM is also a highly experienced qualitative researcher and JT highly experienced in the subject area, so they were able to gain as much nuance as possible from the transcripts [22]. In addition, a third author, TM, an Indigenous community member, ensured all authors were representing the cultural aspects of the themes as accurately as possible.

### Usage Data Automatically Downloaded From the Trial

The iBobbly app was developed with the challenges of remote and very remote regions in mind [12-14]. Internet connectivity was not required to use the app, thereby enabling participants living remotely without a mobile phone network to participate. A Samsung Galaxy 7 tablet with the app pre-installed was provided to participants, removing the need to use a personal mobile phone. When connected to the internet — either during or after the trial — usage data were automatically sent to and stored in a database at the Black Dog Institute in Sydney. These data were available for 40 participants that participated in the RCT, while data were not collected for the remaining 21 participants for numerous reasons such as tablets with flat batteries or connectivity issues. Data were recorded on a number of domains visited: time spent logged onto the app (derived from each individual login and logout times), time spent on each of the 3 self-assessments, time spent on each of the 3 content modules, and number of times the emergency help button was pressed, which presented emergency telephone numbers.

SPSS version 25 was used to conduct regression analyses on 3 measures: suicidal ideation, depression, and psychological distress. Suicidal ideation was measured with the Depressive Symptom Inventory – Suicidality Subscale (DSI-SS), depression with the Patient Health Questionnaire 9 (PHQ-9), and psychological distress with the Kessler Psychological Distress Scale (K10). We sought to examine if time spent on the app led to improved scores on these outcomes. 

## Results

Of the 13 participants interviewed and surveyed, all were of Indigenous background, and 10 identified as female; they had a mean age of 24.15 years (SD 4.7 years). The age range was 19-29 years. More than half (7/13, 54%) were either in full-time or part-time employment, and 46% (6/13) were engaged in either full-time or part-time study.

### Survey Data: General Internet and Technology Use

All participants spent time online every day in a typical week, with a mean of 4.0 hours per day reported (range: 30 minutes to 10 hours). Less time was reported for weekend use, with an average of 3.36 hours (range: 0 minutes to 9 hours). The most popular times for online activity were the afternoon and evening (from 3 pm onwards), with the least activity occurring before midday. All participants went online after 11 pm at least 1 day per week, and 31% (4/13) went online after 11 pm at least 3 days per week.

Smartphones were by far the device of choice for online access, with 77% (10/13) using one daily. No participants used a desktop computer, 54% (7/13) used a tablet, 23% (3/13) used a laptop, and 15% (2/13) used gaming technologies or consoles to go online. The majority reported they would miss their smartphone more than any other technology if they no longer had access to it (9/13, 69%), compared to 23% (3/13) who reported that they would miss their television most. Responses by 85% (11/13) indicated that they accessed the internet via their smartphone or tablet. This compared to 15% (2/13) who accessed the internet at work or in a public place such as a library or internet cafe. The most popular was the use of social networking sites or apps such as Facebook (11/13, 85%). Of the respondents, 69% (9/13) used the internet for email, 61% (8/13) for watching or downloading video clips or movies, and 46% (6/13) to access health information or for gaming.

In relation to the iBobbly app trial, 77% (10/13) of participants interviewed were informed about the trial by a fellow Indigenous community member and 23% (3/13) by a non-Indigenous person (health worker/researcher). All 13 participants stated they would recommend the app to others, and 92% (12/13) stated that they would take part in a similar trial again.

### Usage Data

#### App Usage: Total Time

The usage data of 40 participants were analyzed. The average total time spent using the app was 73.7 minutes, with a range between 8 minutes to 5 hours 42 minutes (over a 6-week period). Of the 15 participants who used the app for more than 1 hour, 4 participants used it for more than 2 hours in total, and 1 participant used it for more than 5 hours.

#### App Usage: Number of Logins

The average number of logins (visits to the app) among the 40 participants was 12.4, with a range between 1 and 43 (over a 6-week period). The average duration of a session using the app was 5.94 minutes. Of the participants for whom there were data, 85% (34/40) completed all 6 activities on the app.

### Regression Analyses

Regression analyses were conducted using SPSS version 25 to examine a potential relationship between time spent on the app and improved outcomes (a dose-response relationship). There were no significant relationships between usage time and any of the 3 outcomes analyzed (suicidal ideation, depression, and psychological distress) for the intervention group (n=18); however, all associations were in a positive direction.

The primary outcome, suicidal ideation, was measured with the DSI-SS: The impact of usage time on reducing scores on this measure was minimal. For every minute spent on the app, DSI-SS scores would reduce by .013 points (R^2^=.29). This means that, for 2 hours spent on the app, DSI-SS scores could reduce by over 1.5 points. The DSI-SS records scores on a range of 0-12, with a score of zero indicating no suicidality and a score ≥2 indicating a risk of suicide in the general population.

High levels of both depression and psychological distress were recorded for this intervention group at baseline, as reported in the trial results [12,13]. Psychological distress was measured with the K10, and for every minute spent on the app, K10 scores would reduce by .007 of a point (R^2^=.35). In simpler terms, it would require 2.5 hours of app use to potentially result in a reduction of just of over 1 K10 point. The K10 records scores on a range of 10-50. Depression was measured using the PHQ-9, and although still in a positive direction, for this small sample, for every minute spent on the app, PHQ-9 scores would reduce by .001 points (R^2^=.268). The PHQ-9 records scores on a range of 0-27.

What the analyses did show was that participants with higher needs (higher baseline and follow-up scores on the psychological measures) used the app more frequently.

### Thematic Analysis

The researchers were investigating to what extent participants found the iBobbly app acceptable, culturally appropriate, and therapeutically effective. Two researchers (KM, JT) identified and agreed upon 3 themes from the interview transcripts: (1) acceptability — being private and acceptable, (2) cultural appropriateness and future help-seeking, and (3) helping with feelings and creating distractions. These themes are explored in detail in the next sections.

#### Acceptability — Being Private and Acceptable

Perhaps not surprisingly given that this was an important part of the interview, exploration of acceptability was a dominant theme in the narratives. Many participants gave brief responses acknowledging their belief that the app was acceptable: “good” (19-F-1, 18-F-3, 29-F-5, 28-F-1, 23-F-13), “very good” (27-M-2, 19-M-6), “great” (21-F-9), or “fantastic” (33-F-7). In comparison, other participants gave longer responses indicating the app was acceptable because it provided a service that was accessible when they needed it:

Accessible when you feel like shit and you need to find a way to de-stress. It’s very helpful.

27-F-11

Indeed, accessibility was important when participants spoke about times when they would not have been able (either physically or psychologically) to access a professional health care service, even if they were willing to do so. Some may have felt known in a small community or simply hesitant to engage a service because they felt uncomfortable. The app allowed them a choice in health care that was previously unavailable:

...accessibility and private… Definitely overcomes the stigma of being known as a client/opting into a service provider… Not everyone is interested in seeing service providers… Encourages you to have a conversation with yourself.

23-F-13

Indeed, the privacy offered by the app was valued because it was “less worrying than actually talking to someone” [27-M-2]. The ability to interact with the app privately, without anyone else needing to be present, meant that youth who may have been reluctant or afraid to speak to family members or health care professionals in a face-to-face setting could still access support:

...because it’s shame to talk... embarrassed to ask for help. Family support not as strong as others. Can confide in iBobbly instead of family.

27-F-11

...it’s non-judgmental. Biggest stigma and shame is being judged by a counsellor or anyone. Can sit in a room on your own and do it.

26-F-8

However, participants also explored ideas around the app’s acceptability in terms of potential negative impacts. 26-F-8 felt that, after a person initially accessed support, the app “got repetitive.” In this way, there was a sense that the app was not harmful, despite its sensitive content, but that people “might get frustrated with it but not suicidal” (26-F-8). One way to potentially mitigate such frustration was to refine iBobbly so it was “…longer, have more content” [27-M-2]. Indeed, 23-F-13 highlighted an important consideration when creating an app that is accessible to vulnerable people who may not have access to any other support — an app may simply not be enough in the face of strong emotions:

If really remote [users] may feel isolated, may feel they have no one to confide in. Risk of invoking feelings, no follow up.

23-F-13

Other participants shared similar concerns, although they were positive about the role of the app in the broader picture of mental health care:

But might over-rely on the app and not see people. Good, but just one of the steps to getting help.

27-M-2

After using app, you are more comfortable answering questions, which may make it easier to talk face-to-face down the track.

28-F-10

#### Cultural Appropriateness and Future Help-Seeking

Closely related to the theme of acceptability, participants’ references to their culture spoke to their views that the app was culturally appropriate. There was a sense that the app overcame the participants’ concerns about a lack of privacy or negative judgment that could often be connected to accessing traditional face-to-face services. In line with this, one participant spoke about “shame” connected to both feeling suicidal and seeking help. This term — which may encompass shame, embarrassment, and a fear of judgement — is often used by young Indigenous people to describe how they are feeling:

Young Aboriginal people have shame factor, so don’t have to talk to people, just use the app. [Might not want to talk to someone because of] privacy and confidence or feeling horrible about something.

19-F-1

Indeed, there was a consistent belief that the app provided support that did not judge the person using it, which created a safe space:

Gives positive and clear way. Non-judgmental education.

33-F-7

“The look and feel” of the app and its original multimedia creations were appreciated as culturally relevant and “right.” This created a safe space for Indigenous people to explore mental health coping strategies without the shame factor:

Really like the voice overs and story sharing…

27-M-2

… down to earth … right wording, all comes down to that and easy, straight-forward, says the right things, images right.

29-F-5

Further, 25-F-4 compared the app favorably to a cognitive behavioral therapy activity she had previously undertaken:

App might be more attractive than a mood & thought table.

25-F-4

Indeed, the images, videos, and voiceovers were not only culturally appropriate but also inclusive to participants’ other needs:

Visually good – I have dyslexia.

23-F-13

In addition, the app could also create a bridge to future help-seeking. Some participants could see the benefit of using the app as an educational tool that improved mental health literacy, thereby facilitating improved cross-cultural communication with mental health professionals:

…Sometimes you can’t find the words. Hearing other stories, makes it easier to find those words. Sometimes you say nothing to clinicians, but the app educates [on the] language to use.

27-M-2

… might work for them [other Indigenous youth] if other things are not working and they are getting frustrated trying to explain their situation and how they are feeling. Maybe use the app when trying to talk to professionals [bring app to appointments].

27-M-2

Finally, the broader research process was positively viewed by 1 participant who regarded the face-to-face interactions of the trial to be useful:

Actual process too, has been mini little counselling sessions for me too. Good for me to have that opportunity.

29-F-5

#### Helping With Feelings and Creating Distractions

Participants’ narratives also spoke to the therapeutic effectiveness of using the app:

It should help — when I was doing it, it reduced all for me. Feelings came and went, but not strong ones. Calmer feeling, wasn’t thinking about bad things.

19-M-12

The psychoeducational features of the app were highlighted and referenced, as they allowed participants to feel distracted from their thoughts and reduce their distress:

...good way to distract yourself. Educates you on where you are at… Better at understanding their feelings and breaking it down, rather than being all over the place.

18-F-3

Was helping me a bit. Did help me get stuff off my mind. Where you sort the fruit out (this referred to a psychoeducational activity included within the app).

19-M-12

Other participants spoke about how the app was helping them to identify their potentially harmful behaviors such as suicidality and even at times, prevent them by changing their perspectives and demonstrating other coping strategies:

It did help me at times, in those moments, dark, suicidal moments, distract me…Kept going through the activities, till suicidal thoughts are changed to better ones. I continue to use it regularly.

29-F-5

…helped me push away my suicidal tendencies. Not completely but helped you settle down and settle the thoughts down and let it out without actually speaking to someone. When using it, I was stressed out, ex-prison stuff, I just jumped on and got some form of release. Helped me from going off — I could feel I was going to lose it — so I would jump on iBobbly. Instead of impulsively drinking or being destructive or having depressing thoughts, I went onto iBobbly.

27-F-11

In line with the idea of providing a distraction or a brief intervention, the app seemed able to change such perspectives and behaviors by providing an interruption for users to give them space to improve their mental state and decision making:

It gives a buffer, has numbers, gives an opportunity to ask for help.

33-F-7

...instead of smashing a window or something, you can pick up the iBobbly or have a scream at it or something. Definitely, the suicidal thinking can calm someone down, gives options instead of suicide.

26-F-8

There was also a sense that the app’s activities helped to improve self-awareness and interpersonal communications, which also spoke to ideas around improving cross-cultural communication:

…even to recognize that you are not quite right … encourages you to have a conversation with yourself. Activities to improve behavior and interactions with other people.

23-F-13

However, 23-F-13 wondered whether some of the activities were repetitive and suggested that people should be able to access more advanced tools when needed:

Looks simplistic, may devalue the messages. Didn’t allow advancement to a more advanced set of questions. You need to challenge. Was basic, aimed at younger, repetitive.

23-F-13

In this way, the app’s therapeutic effectiveness may have been influenced by the participants’ therapeutic needs at the time of use.

Finally, there was also an indication that the app was therapeutically effective because it could travel with the participants; it did not matter where they were when they needed support. In this way, the app could offer support that was both consistent and appropriate, something that would not have been otherwise accessible:

If you use the app when distressed and impulsive; then, that reduces the depression and suicidal thinking. I took it to [location away from home]; shit things happened, and it helped.

19-F-1

This may indicate the usefulness of the app for brief interventions rather than for intensive therapeutic use. 

## Discussion

### iBobbly Usage and Therapeutic Impact

Participants used the iBobbly app for over 73 minutes on average, with over 12 logins on average over the 6-week trial. This is an encouraging indication of this app’s capacity to engage users considering the challenges in engaging users of mental health apps, as previously reviewed [23]. The intervention protocol did not include encouragement, support, or weekly reminders from a clinician, all previously reported to improve adherence in low-intensity eHealth interventions [23]. Rather, the iBobbly app was purely self-directed and relied solely on user motivation to logon and complete activities. It is questionable how much therapeutic value could be derived from 73 minutes of self-assessments and therapeutic activities; however, this usage is comparable to the effective ultra-brief acceptance and commitment therapy sessions of one or two 20-30-minute sessions delivered in primary care settings by Strosahl et al [24]. Further, 2 female participants (19-F-1 and 27-F-1) gave examples where the app helped during times of suicidal thinking. This demonstrates that even a short amount of time on the app can be effective when the content is appropriate and easily accessible.

Participants returned to reuse the app regularly, on average over 12 times, although it is difficult to determine the therapeutic effectiveness of each session or the cumulative effect of each session. Though participants completed identical activities, these activities allowed for choice, and repeat visits to the app highlight that it managed to maintain participant engagement. The average duration of a session using the app was quite brief, thereby indicating that the intervention at the very least provided some distraction from distressing thoughts, feelings, or activities. Participants found app use helped “push away my suicidal tendencies,” and it was a “good way to distract yourself. Educates you on where you are at.” These patterns of multiple brief interventions may suggest the usefulness of the app and similar interventions [25] as an adjunctive treatment rather than a standalone measure. This was reinforced in the interviews, with participants regarding the app as “one of the steps” to getting help. The app helped give participants language to their experiences, as well as identify more critically stressful times in their lives and coping strategies that could be effective. Consequently, the app was a tool that could be used during consultations with health professionals to improve mental health literacy and communication, thereby helping overcome shame. While it is likely that non-Indigenous people with mental health issues also suffer shame, the cultural aspects of racism in the health care system for a disadvantaged minority such as Indigenous Australians may exacerbate their feelings of shame.

The regression analyses completed on 3 outcome measures, suicidal ideation, depression, and distress, showed just a minimal indication that increased app use would lead to improved outcomes. With a small sample of 18 participants and usage data included from just the intervention group, the analyses lacked power and precision. The analyses did, however, show that participants with higher needs (higher baseline and follow-up scores on the psychological measures) used the app more frequently. Their scores post-trial were reduced but still elevated, again suggesting the app’s suitability as an adjunctive treatment particularly for users with high therapeutic needs. The regression analyses contrast with the qualitative results and the significant reductions in depression and anxiety as reported in the RCT results; however, this may be explained by the previous use of a larger sample size of 61 participants [13,26]. Therapeutic effectiveness was identified as 1 of 3 themes in the thematic analysis, with participants considering the app to have multiple and diverse therapeutic features. Distraction was referred to by participants, aligning with the brief nature of their engagements. It is also possible they were not interested in longer sessions due to the “repetitive” nature of some activities or simply due to time restrictions, thereby engaging with the app in the brief and typical way young people may engage with any app. More enduring therapeutic value was also identified, such as perspective gaining and reduced impulsivity, calmer feelings, and improved interpersonal relationships. In summary, while symptom improvement was not captured by the impact of time spent on the app, the app was viewed as therapeutic, and this aligns with the previously reported significant reductions in depression and distress [13].

### Acceptability and Cultural Appropriateness

The analyses indicate that participants viewed the iBobbly app as an acceptable intervention for themselves and their community members. Indeed, a notable feature of this trial was a very low attrition rate of 3%, as reported previously [13]. This remarkably low dropout rate may indicate the acceptability of the app for the majority of participants, a view supported by the findings of the current study. Of interest was how participants were informed about the trial and whether being informed by other Indigenous community members would increase the likelihood of signing up. It is often assumed that local support for an initiative will improve adoption and that word-of-mouth is an effective method for Indigenous community members to share either positive or negative views on a health intervention. This was evident in this trial, with a large majority of participants (77%, 10/13) referred by other Indigenous community members acting as key influencers [13]. This demonstrates a positive view of the app’s acceptability and cultural appropriateness, as does the willingness of all participants in this analysis (n=13) bar 1 to potentially take part in another similar trial in the future. In addition, all participants surveyed (n=13) were willing to recommend similar trials to others.

App trials have shown favorable results on adherence even above desktop-based interventions [27]. In addition, interventions delivered within the structure of RCTs rather than open access also improved adherence [28]. The iBobbly intervention followed this pattern, with participants describing benefit from the flexible nature of using a mobile device as well as iBobbly app adherence benefitting from the person-to-person follow-ups and rigor of an RCT. The 2015 study by Povey et al [14] evaluated 2 mHealth interventions for Indigenous community members: the iBobbly app as detailed in this paper and the AimHi mHealth app designed for health workers. The study by Povey et al [14] found that eHealth interventions, such as the iBobbly and AimHi apps, are more likely be effective and acceptable for Indigenous communities if they are designed in partnership with community members and use appropriate language and imagery [14]. The iBobbly participants’ recommendations for improving future versions of the app demonstrated their interest in helping to create optimal interventions in the suicide prevention space for their own people. This feedback, coupled with the positive comments around acceptability, indicated that Indigenous youth (under 25 years old) viewed the app as both acceptable and culturally appropriate.

### Conclusion

Both RCTs and eHealth interventions are extremely rare in Indigenous communities, particularly when targeting suicidal ideation. Consequently, an accurate understanding of participants’ engagement with such interventions is crucial. This analysis showed Indigenous youth are heavy consumers of internet and communications technologies and that their engagement with the iBobbly app demonstrated an interest in utilizing mHealth for suicide prevention and social and emotional wellbeing. Participants considered the iBobbly app to have therapeutic value and to be an acceptable and culturally appropriate tool for themselves and their wider communities. Participants reported improvements in psychological wellbeing, increases in mental health literacy, and reductions in shame, which they attributed to the iBobbly app use. It is most likely that effective research partnerships are key components in the successful implementation of mHealth apps such as iBobbly for Indigenous community members. Further research where the iBobbly app is available as a standalone app available for download will demonstrate its power to engage users in real-world settings.

## Appendix

Multimedia Appendix 1 Measures of Technology Use survey developed by the Young and Well Cooperative Research Centre.

Multimedia Appendix 2 iBobbly semistructured interview.

## Abbreviations

DSI-SS: Depressive Symptom Inventory – Suicidality Subscale
K10: Kessler Psychological Distress Scale
mHealth: mobile health
PHQ-9: Patient Health Questionnaire 9
RCT: randomized controlled trial
SEWB: Social and Emotional Wellbeing
