# Supplementary material for: Usage and Acceptability of the iBobbly App: Pilot Trial for Suicide Prevention in Aboriginal and Torres Strait Islander Youth
Source: JMIR Ment Health. 2020 Dec 1;7(12):e14296. doi: 10.2196/14296 (PMC7738247; doi:10.2196/14296)
Supplement: Multimedia Appendix 1 [file mental_v7i12e14296_app1.docx]

Measures of Technology Use (YAWCRC)

| **How often do you use the internet or spend time online?**  1 = Every day or almost every day 2 = Once or twice a week 3 = Once or twice a month 4 = Less than once a month |
| --- |
| **Approximately how much time would you spend online or using the internet on a normal (weekday/workday)?** 1. HOURS PER DAY given (specify_______) (RANGE 0.25 TO 24) 2. MINUTES PER DAY given (specify________) (RANGE 1 TO 120) |
| **Approximately how much time would you spend online or using the internet on a normal (weekend/non-work day)?** 1. HOURS PER DAY given (specify_______) (RANGE 0.25 TO 24) 2. MINUTES PER DAY given (specify________) (RANGE 1 TO 120) |

**When are you most active online (using, for example, a mobile phone, computer or tablet to communicate with others, look for information or to entertain yourself) on a typical school or work day?*****

1 = early morning (5am - 9am)

2 = mid-morning (9am - 12noon)

3 = early afternoon (12noon - 3pm)

4 = mid-afternoon (3pm - 6pm)

5 = evening (6pm - 11pm)

6 = night time (11pm - 5am)

|  |
| --- |
| **Which of the following technologies do you use on a more or less daily basis? (please choose as many as apply to you)**  1 = A smart phone  2 = A mobile phone  3 = A tablet (eg: iPad)  4 = A laptop  5 = A desktop computer  6 = A games console or portable gaming device (eg. Playstation, Xbox, Wii, PSP, DS, Gameboy)  7 = other handheld portable devices (eg. MP3 player, iPod Touch)  8 = A television  9 = other  10 = none of the above |
| **Which of the following technologies would you miss the most if you no longer had access to it? (please choose one)**  1 = A smart phone  2 = A mobile phone  3 = A tablet (eg: iPad)  4 = A laptop  5 = A desktop computer  6 = A games console or portable gaming device (eg. Playstation, Xbox, Wii, PSP, DS, Gameboy)  7 = other handheld portable devices (eg. MP3 player, iPod Touch)  8 = A television  9 = other  10 = none of the above |
| **Where do you most commonly access the internet/go online?**  1. Anywhere via my smart phone or tablet  2. At a friend’s home  3. At a relative’s home  4. In living room (or other public room) at home  5. In own bedroom (or other private room) at home  6. School, TAFE or university  7. Work  8. Other public place (eg. library, shopping centre, café, internet café)  9. Other (specify)  999. Don’t know  888. Refused |
| **Please choose from the following list all the things you have done online in the PAST MONTH?**  1 = Accessed chatrooms  2 = Accessed health information  3 = Accessed online virtual worlds (e.g. Second Life)  4 = Accessed social network websites (e.g. Facebook)  5 = Checked email  6 = Gambled  7 = Listened to, downloaded or uploaded music (e.g. iTunes, Spotify, Songle)  8 = Made or received Voice Over Internet Protocol (VOIP) phone calls (e.g. Skype)  9 = Used online or email counselling  10 = Played games alone  11 = Played games with others over the internet  12 = Posted or viewed photos (e.g. Flickr, dropshots, pinterest, Instagram)  13 = Read a blog entry  14 = Read or watched the news  15 = Searched for new friends  16 = Used a webcam  17 = Used an instant messenger (e.g. MSN or gmail messenger)  18 = Used eBay, auction sites, Internet shopping facilities  19 = Used forums, bulletin boards, or discussion groups  20 = Used the Internet for school, study or work  21 = Used Twitter  22 = Watched, downloaded or uploaded video clips, cartoons, movies, etc, e.g. YouTube  23 = Written a blog or online diary  24 = Accessed pornography  25 = Sexted (sent or received nude or semi-nude photos of yourself or others)  24 = Something else (specify_____)  25 = (None) |
| **How true are the following statements for you? Response options:**  **1. Not true**  **2. A bit true**  **3. Very true**  1. I find it easier to be myself when online than when I am with people face-to-face  2. I talk about different things with people when online than I do when face-to-face  3. When I am online, I talk about private things that I do not share with people face-to-face  4. I go online much more on the weekends than I do on a regular school or work day [optional question]  5. When I am going through a difficult time, I go online less often  6. When I am going through a difficult time, going online makes me feel better |
| **How many days a week do you go online after 11pm at night?**  1 = 0  2 = 1  3 = 2  4 = 3  5 = 4  6 = 5  7 = 6  8 = 7  999. Don’t know |
