# Supplementary material for: Usage and Acceptability of the iBobbly App: Pilot Trial for Suicide Prevention in Aboriginal and Torres Strait Islander Youth
Source: JMIR Ment Health. 2020 Dec 1;7(12):e14296. doi: 10.2196/14296 (PMC7738247; doi:10.2196/14296)
Supplement: Multimedia Appendix 2 [file mental_v7i12e14296_app2.docx]

iBobbly Semi Structured Interview

(1) What are your thoughts in general on the iBobbly app?  *(acceptability & cultural appropriateness)*

(2) Do you think using the app might help Aboriginal young people? *(acceptability & cultural appropriateness)*

If YES, in what way?

If NO, why not?

(3) Do you think using the app might make things worse for Aboriginal young people? *(acceptability & cultural appropriateness)*

If Yes, in what way? If No, why not?

(4) Do you think this a suitable app for Aboriginal young people to try to help them reduce; *(effectiveness)*

(a) Suicidal thinking

(b) Depression

(c) Distress

(d) Impulsivity

(5) Do you think using the app encourages people to take more responsibility for themselves and their lives? If Yes, in what way? If No, why not? *(effectiveness)*

(6) Do you think the app might increase help-seeking behavior in Aboriginal young people *(effectiveness)*

(7) Did the app help you? *(effectiveness)*

No, why not? Yes, how?

(8) Why did you decide to take part in this study? *(participant motivation)*

(9) How did you find out about it? *(referral processes)*

(10) If someone informed you about it what is their cultural background? *(participant motivation)*

(11) Would you recommend others to try the app out, why, why not? *(acceptability & cultural appropriateness)*

(12) Now that you have finished this trial, would you be more willing or not to get involved in other similar studies? *(acceptability)*
